# Prospective randomized clinical studies involving reirradiation: update of a systematic review

**DOI:** 10.1007/s00066-023-02118-1

**Published:** 2023-07-27

**Authors:** Carsten Nieder, Jonas Willmann, Nicolaus H. Andratschke

**Affiliations:** 1 Department of Oncology and Palliative Medicine, Nordland Hospital Trust, 8092 Bodø, Norway; 2 Department of Clinical Medicine, Faculty of Health Sciences, UiT—The Arctic University of Norway, 9038 Tromsø, Norway; 3 Department of Radiation Oncology, University Hospital Zürich, 8091 Zurich, Switzerland

**Keywords:** Radiotherapy, Radiation oncology, Radiation retreatment, Reirradiation, Randomized studies

## Abstract

**Background:**

Reirradiation is a potentially useful option for many patients with recurrent cancer, aiming at cure or symptom palliation, depending on disease/recurrence type and stage. The purpose of this follow-up study to a previous review from 2016 was to summarize all recently published randomized trials. Points of interest again included identifcation of methodological strengths and weaknesses, practice-changing results, and open questions.

**Material and methods:**

Systematic review of trials published between 2015 and February 2023.

**Results:**

We reviewed 7 additional trials, most of which addressed reirradiation of head and neck or brain tumours. The median number of patients was 60. Mirroring the previous review, trial design, primary endpoints and statistical hypotheses varied widely. The updated results only impact on decision making for reirradiation of nasopharynx cancer and glioma. Patients with one of these diseases, as well as other head and neck cancers, may benefit from reirradiation-induced local control, e.g. in terms of progression-free survival. For the first time, hyperfractionated radiotherapy emerged as preferred option for recurrent, inoperable nasopharynx cancer. Despite better therapeutic ratio with hyperfractionation, serious toxicity remains a concern after high cumulative total doses. Randomized trials are still lacking for prostate cancer and other sites.

**Conclusion:**

Multicentric randomized trials on reirradiation are feasible and continue to refine the current standard of care for recurrent disease after previous radiotherapy. Ongoing prospective studies such as the European Society for Radiotherapy and Oncology and European Organisation for Research and Treatment of Cancer (ESTRO-EORTC) observational cohort ReCare (NCT: NCT03818503) will further shape the clinical practice of reirradiation.

## Introduction

**Fig. 1:**
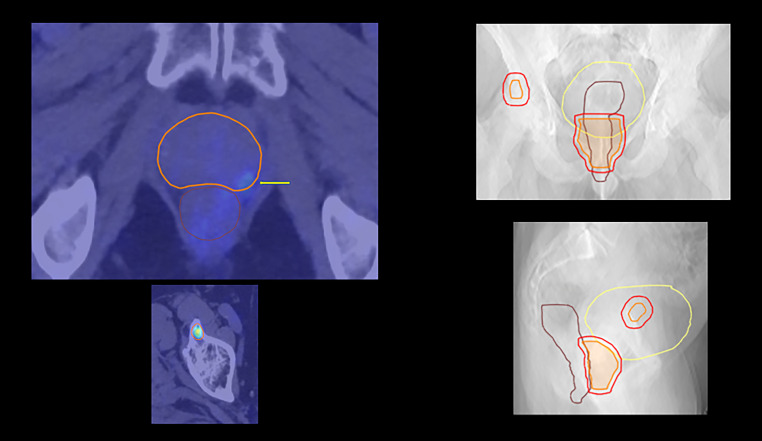
Type 1 reirradiation is a new course of radiotherapy that has geometrical overlap with the irradiated volume of previous courses (left upper panel: prostate reirradiation to a biopsy-confirmed ^68^Ga-PSMA positron emission tomography (PET) positive (*yellow arrow*) relapse after previous external beam radiotherapy), and type 2 is a new course with concerns of toxicity from the cumulative doses but in which there is no overlap with the irradiated volume of previous courses (other panels, treatment planning computed tomography with fused PET in the same patient). Irradiating the single bone metastasis might increase the risk of bladder or bowel toxicity. In the absence of toxicity concerns, the term “repeat organ irradiation” is recommended. *CTV* clinical target volume: *orange*, *PTV* planning target volume: *red*, rectum: *brown*, bladder*: yellow*

Throughout many decades of clinical research and radiobiological animal studies, reirradiation has evolved into a widely utilized treatment, e.g., for bone metastases, brain metastases, head and neck cancer, prostate cancer and other malignancies [1–7]. A recent consensus endorsed by the European Society for Radiotherapy and Oncology (ESTRO) and the European Organisation for Research and Treatment of Cancer (EORTC) aimed at a standardized classification of different forms of reirradiation and reporting [8]. The use of this classification in daily clinical practice and ongoing research will facilitate accurate understanding of the clinical implications of reirradiation and allow for cross-study comparisons. The consensus document was based on an adapted Delphi process and a systematic review of the literature. Reirradiation is a new course of radiotherapy, either to a previously irradiated volume (irrespective of concerns of toxicity) or where the cumulative dose raises concerns of toxicity [8]. Type 1 is a new course of radiotherapy that has geometrical overlap with the irradiated volume of previous courses (Fig. 1), and type 2 is a new course with concerns of toxicity from the cumulative doses but in which there is no overlap with the irradiated volume of previous courses, e.g., in the lungs. Repeat organ irradiation is a new course of radiotherapy to a previously irradiated organ but without overlap of the irradiated volumes and without concerns for toxicity from cumulative doses. As consistently reported, reirradiation might provide worthwhile clinical benefit in terms of symptom palliation, local tumor control and sometimes even cure after diagnosis of local or regional relapse or second primary tumours in a pretreated region. In parallel to single-arm studies, randomized clinical trials have been performed [9–17]. A previous review from 2016 evaluated the published randomized trials in order to identify methodological strengths and weaknesses, comment on the results, clinical implications and open questions, and advise on the planning of future trials [18]. The present updated review examines all additional publications up to February 2023.

## Methods

Inclusion was limited to trials published between 2015 and 2023, i.e. the recent literature. Trials were identified through systematic searches of the databases PubMed, Scopus and Web of Science by use of the key words ‘reirradiation’, ‘re-irradiation’, ‘repeat radiotherapy’, ‘radiation retreatment’ and ‘recurrent AND radiotherapy’ in February 2023. References from published trials and the consensus document were cross-checked.

## Results and discussion

We identified and reviewed 7 randomized trials, which are presented in Table 1, together with 9 already discussed trials. Most of these new publications were related to head and neck tumours (two Chinese trials on nasopharynx cancer [19, 20] and one French trial on head and neck squamous cell cancer [21]) or glioblastoma/high-grade glioma (*n* = 3) [22–24]. The median number of patients in these 7 trials was 60. Three trials had a standard arm without reirradiation (surgery vs. reirradiation for nasopharynx cancer [19]; chemotherapy vs. fractionated stereotactic radiosurgery with chemotherapy for glioma [22]; bevacizumab vs. bevacizumab and reirradiation for glioblastoma [24]). The trials addressed important questions regarding dose/fractionation, combination with anti-cancer drugs, and toxicity.

**Table 1:** Randomized studies 2000–2023: disease site, methods, population and main results

Author and year of publication	Disease site	Study type, inclusion	Arms, design, endpoint, statistics	Patient number and characteristics	Median follow-up	Results and comments
Li et al. 2006 [9]	Nasopharynx cancer	Single centre dose escalation, China, 1999–2002	54 Gy followed by 16, 20 or 24 Gy in 4‑Gy fractions (3 fractions per week) 4 primary endpoints, power/assumed differences not reported	36, interval ≥ 6 months, N0 M0	27 mo	In each arm 2–3 patients had received induction chemotherapy 3‑year recurrence-free survival was best in the 24-Gy boost group, *p* = 0.047 Similar OS, *p* = 0.6 Similar acute and late toxicity rates, but one fatal bleeding event in the 24-Gy boost group, which also had higher incidence of trismus, *p* = 0.08
Tian et al. 2014 [10]	Nasopharynx cancer	Single centre phase 2, China, 2003–2007	IMRT 68 Gy in 34 fractions vs. 60 Gy in 27 fractions Overall survival, 80% power to detect 23% difference	117, KPS ≥ 70, interval > 6 months	25 mo	Longer OS in the 60-Gy arm, *p* = 0.06 Similar PFS Less mucosal necrosis in the 60-Gy arm, *p* = 0.02
Guan et al. 2016 [11]	Nasopharynx cancer	Single centre phase 2, China, 2002–2008	IMRT 60 Gy in 27 fractions alone vs. same RT + concomitant weekly cisplatin Overall survival, 80% power to detect 30% difference	69, KPS ≥ 70, interval > 6 months	35 mo	Longer OS in the combined modality arm, *p* = 0.049 No significant increase in late toxicity, but more haematologic toxicity in the combined modality arm
Liu et al. 2021 [19]	Nasopharynx cancer	Three centres, phase 3, China, 2011–2017	Endoscopic nasopharyngectomy or IMRT 60–70 Gy (2–2.36 Gy per fraction, 5 fractions per week) Overall survival, 80% power and a two-sided 5% significance-level hazard ratio of 0.52	200, KPS ≥ 70, ≥ 12-month disease-free interval between the initial course of radiotherapy and recurrence, age 18–70 years	56 mo	Improved 3‑year overall survival after surgery (86% versus 68% in the IMRT group, *p* = 0.0015)
You et al. 2023 [20]	Nasopharynx cancer	Three centres phase 3, China, 2015–2019	IMRT 60 Gy in 27 fractions vs. 65 Gy in 54 fractions (2 fractions per day) Overall survival and severe late complications, 80% power to detect 20% difference (survival) and 24% difference (toxicity grade 3 or more)	144, KPS ≥ 70, interval > 12 months, age 18–65 years, no radiation-induced complications grade ≥ 3	45 mo	Reduced grade 3 or worse late radiation-induced toxicity in the hyperfractionation group (34% versus 57%, *p* = 0.02) Better 3‑year overall survival after hyperfractionation (75% versus 55%, *p* = 0.01) 49% of patients in the hyperfractionation group and 46% in the standard fractionation group had locoregional relapse significant differences favouring hyperfractionated radiotherapy in the general quality-of-life domains of global health status, role functioning, and social functioning, and in the symptom burden domains of pain, financial difficulties, and loss of appetite
Janot et al. 2008 [12]	Head and neck squamous cell carcinoma	Multicentre phase 2, France/Belgium, 1999–2005	6 cycles of postoperative RT, each with 5 fractions of 2 Gy, with concomitant 5‑FU and hydroxyurea (9 day rest period between cycles) vs. observation DFS at 3 years, 80% power to detect 20% difference	130, KPS ≥ 80, interval to salvage surgery ≥ 6 months, macroscopically complete resection, no severe sequelae after initial course	Not reported	Longer DFS in the RT arm, *p* = 0.006 Better locoregional control, *p* < 0.0001 3 treatment-related deaths within one month after RT and 2 at later time points More grade 3 or 4 late toxicity after RT, *p* = 0.06 Similar OS, *p* = 0.5
Tortochaux et al. 2011 [13]	Head and neck squamous cell carcinoma	Multicentre phase 3, France, 1999–2005	6 cycles of RT, each with 5 fractions of 2 Gy, with concomitant 5‑FU and hydroxyurea (9 day rest period between cycles) vs. weekly methotrexate Overall survival at 1 year, 80% power to detect 19% difference	57 (premature closure, 160 required), KPS ≥ 70, unamenable to curative salvage therapy, interval ≥ 6 months, no severe sequelae after initial course	Complete, all patients died	Similar OS, *p* = 0.6 4 complete responses after radiochemotherapy vs. none after chemotherapy alone More toxicity after radiochemotherapy, including grade 5 events
Rudžianskas et al. 2014 [14]	Head and neck squamous cell carcinoma	Single centre phase 2, Lithuania, 2008–2011	EBRT 50 Gy in 25 fractions vs. HDR BT 30 Gy in 12 fractions Statistical hypothesis and primary endpoint not reported	64, KPS ≥ 80, no grade 3 or 4 toxicity from initial course	Not reported	Significantly smaller PTV in BT arm despite randomization BT was associated with better LC, *p* < 0.001, OS, *p* = 0.002, and late toxicity, *p* = 0.001
Tao et al. 2018 [21]	Head and neck squamous cell carcinoma	Multicentre phase 2, France, 2010–2014	60 Gy over 11 weeks with concomitant 5FU—hydroxyurea vs. 60 Gy (1.2 Gy twice daily) over 5 weeks with cetuximab Primary endpoint was treatment interruption > 15 days (acute toxicity) Simon’s two-stage design, with alpha = 10% and beta = 10%, 28 subjects were expected in each arm (stage 1 = 9 patients, stage 2 = 19 patients). After evaluation of the 9 first patients, if the number of patients which experienced toxicities was ≥ 3, the study had to be stopped. If this number was ≤ 2, 19 additional patients would be included in each arm	60, PS 0‑1, > 6 months between initial RT and salvage surgery, sufficient healing for beginning reirradiation within 8 weeks of salvage surgery, age 18–75 years, without severe sequelae of initial RT	36 mo	Similar rates of more than 15 days of treatment interruption due to toxicity (*n* = 1 and 0, respectively, *p* = 0.49) Toxicities and DFS were not different between both arms
Chow et al. 2014 [15]	Bone metastases	International phase 3, 2004–2012	8 Gy single fraction vs. 20 Gy (5 or 8 fractions, depending on initial dose and body region) Pain response after 2 months, non-inferiority (difference < 10% with reference to the upper 95% CI of the 8‑Gy arm)	850, minimum interval 4 weeks, no spinal cord compression, pathological fracture or impending fracture, pain score 2–10	12 mo	Intention-to-treat analysis confirmed non-inferiority Per protocol analysis did not confirm non-inferiority Significantly more toxicity after 20 Gy
Wick et al. 2014 [16]	Glioblastoma	International phase 2, 2009–2011	36 Gy alone (2-Gy fractions) vs. 36 Gy + APG-101 weekly until progression PFS at 6 months, optimal two-stage design of Simon	91, adult patients, 1st or 2nd progression, not resectable or residual tumor after resection, largest tumor diameter 1–4 cm, KPS ≥ 60, interval ≥ 8 months	11.4 mo	PFS was significantly better in the combined modality arm
Bergman et al. 2020 [22]	Glioblastoma and high-grade glioma (bevacizumab resistant)	Single centre, United States, 2012–2016	BEV-based chemotherapy with irinotecan, etoposide, temozolomide, or carboplatin. Other arm: EBRT 8 Gy × 4 fractions within 2 weeks to the gross target volume and 6 Gy × 4 fractions to the clinical target volume (fluid-attenuated inversion recovery abnormality) plus BEV-based chemotherapy Primary endpoints: local tumor control at 2 months and PFS 80% power to detect 30% improvement by EBRT (sample size of 76 patients)	35 (closed due to slow accrual), KPS ≥ 70	Not reported	Patients treated with RT had significantly improved PFS (5.1 vs. 1.8 months, *p* < 0.001) and improved LC at 2 months (82% vs. 27%, *p* = 0.002) Overall median survival was 7.2 months with RT vs. 4.8 months with chemotherapy alone, *p* = 0.11
Voss et al. 2020 [23]	Glioblastoma or progression from lower grade glioma	Three centres, phase not stated, Germany, 2013–2017	Dietary intervention over 9 days that consisted of 2 calorically restricted KD 3‑day intervals flanking 3 days of fasting plus reirradiation vs. reirradiation and calorically unrestricted diet Most patients had 5 or 10 fractions of reirradiation (5 × 4 Gy, 10 × 3.5 Gy) Increase of PFS at 6 months from 0% to 30% with a power of 80%	50, KPS ≥ 60, interval at least 6 months	Not reported	No significant difference in PFS at 6 months: 20% vs. 16%, *p* = 0.7 Similar median OS: 331 days vs. 291 days
Tsien et al. 2023 [24]	Glioblastoma	Multicentre phase 2, 2012–2016	Bevacizumab alone vs. Bevacizumab plus reirradiation (10 fractions of 3.5 Gy) Overall survival, 80% power to detect a 31% reduction in the hazard ratio to 0.69 at the significance level of 0.1	170, KPS ≥ 60, interval at least 6 months, recurrent tumor ≤ 6 cm	12.8 mo	No improvement in overall survival for BEV + RT, *p* = 0.46 (median 10.1 versus 9.7 months) Median PFS for BEV + RT was 7.1 versus 3.8 months for BEV, *p* = 0.05
Kouloulias et al. 2003 [17]	Skin metastases from breast cancer after mastectomy and RT	Single centre phase 2, Greece, 1998–1999	PEGylated liposomal doxorubicin and 17 fractions of 1.8 Gy vs. same drug and 8 fractions of 3 Gy + one fraction of 4 Gy Disease-free interval to local relapse, power/assumed difference not reported	30, KPS > 70, superficial tumours	Not reported	Similar efficacy and DFILR, *p* = 0.58 Less acute skin toxicity with 1.8-Gy fractions (all grade 1 or 2), *p* = 0.027 Less late skin toxicity with 1.8-Gy fractions (all grade 1 or 2), *p* < 0.001
Schouten et al. 2022 [25]	Locally recurrent breast cancer	Single centre phase 2, Netherlands, 2010–2019	32 Gy was given in 8 fractions of 4 Gy in 4 weeks, but after January 2015, the regimen was changed to 46 Gy in 23 fractions of 2 Gy, at five fractions per week Hyperthermia was added once a week after radiotherapy The combined arm was treated with four cycles of weekly cisplatin 40 mg/m^2^ 90% power to detect an increase in the local control rate after 1 year from 54% in the standard treatment arm to 69% in the study arm with cisplatin	49 (study closed due to slow accrual), PS 0–2, not suitable for resection	7.1 and 12.6 mo, respectively	Similar complete response rates: 61% each Partial response rate was 30% in the standard arm and 33% in the combined arm, *p* = 0.79 One-year local progression-free interval was 81.5% in the standard arm and 88% in the combined arm, *p* = 0.95 Grade 3 or 4 acute toxicity 25% (standard) and 29% of patients (combined arm), *p* = 0.79

*BEV* Bevacizumab, *DFILR* Disease-free interval to local relapse, *DFS* disease-free survival, *EBRT* external beam radiotherapy, *HDR BT* high-dose-rate brachytherapy, *IMRT* intensity-modulated radiotherapy, *KD* ketogenic diet, *KPS* Karnofsky performance status, *LC* local control, *OS* overall survival, *PFS* progression-free survival, *PTV* planning target volume, *RT* reirradiation

Trial design, primary endpoints and statistical hypotheses varied widely. In three publications from our first review [18], information on these crucial components was missing to some extent. Now, no such missing data were identified. In line with the previous review, several trials were powered to detect substantial differences in overall survival or progression-free survival, i.e. ≥ 20%, which are uncommon in this setting. All reports provided sufficient details on inclusion and exclusion crtiteria allowing the readers to judge these quality criteria. However, the median length of follow-up was not reported in two of the publications [22, 23].

### Nasopharynx cancer

Except for the landmark bone metastases trial from 2014, the randomized phase 3 trial by Liu et al. [19] represents the largest reirradiation study (*n* = 200). These researchers confirmed that salvage surgery (endoscopic nasopharyngectomy) is an important option for resectable recurrences, leading to improved 3‑year overall survival compared to intensity-modulated radiotherapy (IMRT; 86% versus 68%, *p* = 0.0015). Most patients had N0‑1 disease. Seventy-one (71%) of the 100 patients in the IMRT group received cisplatin-based chemotherapy. IMRT was not fully standardized regarding dose per fraction and total dose (60–70 Gy). A proportion of patients (> 30%) had received initial radiotherapy with less advanced techniques that often result in higher doses to critical organs at risk. In line with previous studies, reirradiation caused a relatively high rate of serious toxicity. The most common grade 3 or worse radiation-related late adverse event was pharyngeal mucositis (26% after IMRT). Five (5%) of the patients who underwent surgery and 20% of patients who underwent IMRT died due to late toxic effects specific to radiotherapy. Connecting the toxicity in the reirradiation arm to initial radiotherapy or trial radiotherapy was difficult due to the long-term nature of radiation-related toxicity. However, the fact that high-dose reirradiation may cause fatal toxicity is well-known from the literature [26–28].

A previous randomized trial in nasopharynx cancer had evaluated de-escalation of the equivalent dose (EQD2) for late responding normal tissues, while maintaining the same EQD2 for tumour cells [10]. This was accomplished by selecting a slightly hypofractionated experimental regimen, which was compared to a conventional regimen with 2‑Gy fractions and longer overall treatment time (Table 1). It was assumed that normal tissue sparing would result in less life-threatening toxicity and thus better overall survival, i.e. a better therapeutic ratio. Indeed, the results showed a 5-year overall survival rate of 44% versus 30%, but the difference was not statistically significant (*p* = 0.06) in this underpowered study. Serious toxicity was not uncommon and therefore, further EQD2 reduction has now been studied. Hyperfractionation was employed to mitigate toxicity, while maintaining the same overall treatment time [20]. Standard IMRT was identical to the previous study, i.e. 60 Gy in 27 fractions, while hyperfractionated IMRT featured 65 Gy in 54 fractions (2 fractions per day). Overall survival and toxicity were the primary endpoints in this 144-patient trial. Patients were not allowed to have radiation-induced complications grade ≥ 3 before reirradiation. Regarding outcomes, reduced grade 3 or worse late radiation-induced toxicity was observed in the hyperfractionation arm (34% versus 57%, *p* = 0.02), in line with radiobiological assumptions. Significant differences favouring hyperfractionated radiotherapy were seen in the general quality-of-life domains of global health status, role functioning, and social functioning, and in the symptom burden domains of pain, financial difficulties, and loss of appetite. Furthermore, 3‑year overall survival was better after hyperfractionation (75% versus 55%, *p* = 0.01). Efficacy was suboptimal, given that 49% of patients in the hyperfractionation group and 46% in the standard once-daily fractionation group had locoregional relapse. A possible strategy would be to moderately increase the dose per fraction in the hyperfractionation regimen and/or to add 1–2 additional days, resulting in dose escalation. However, this carries a risk of increasing fatal toxicity and leaving overall survival unchanged. Alternatively, assuming that not all recurrent tumours are sufficiently sensitive to further irradiation, incorporating other approaches such as drug treatment may be warranted [29].

### Head and neck squamous cell cancer

A French group reported a phase 2 randomized multicentric trial comparing two regimens of reirradiation after salvage surgery: mono-fractionated radiotherapy with concomitant chemotherapy and hyperfractionated radiotherapy with cetuximab as experimental arm [21]. The primary endpoint was the comparison of the number of patients with a treatment interruption for more than 15 days, due to acute toxicity. Thus, size was limited to 60 patients. Inclusion criteria included, e.g., > 6 months between the initial course of radiotherapy and salvage surgery, sufficient healing for beginning reirradiation within 8 weeks of salvage surgery, age 18–75 years, WHO performance status (PS) 0–1, no severe sequelae of initial radiotherapy, and > 50% of recurrent tumor had received ≥ 50 Gy during previous irradiation. One arm employed the so-called Vokes protocol, i.e. 60 Gy in 11 weeks (6 cycles, with each cycle delivering 2 Gy/fraction, 5 days/week, with concomitant hydroxyurea (1.5 g/d orally) and continuous infusion fluorouracil (800 mg/m^2^/day), with 9‑day rest periods between cycles (split course)). In the radiobiologically more sound hyperfractionated radiotherapy arm, patients received a total dose of 60 Gy in 50 fractions, 1.2 Gy/fraction, 2 fractions/day, 5 days/week during 5 weeks. Cetuximab was initiated one week before radiotherapy at a loading dose of 400 mg/m^2^, followed by weekly 250 mg/m^2^ during radiotherapy. Similar rates of more than 15 days of treatment interruption due to toxicity were reported (*n* = 1 and 0, respectively, *p* = 0.49). Overall, toxicities and disease-free survival (DFS) were not different between the two arms. Thus, hyperfractionated reirradiation of 60 Gy/5 weeks with cetuximab was tolerable. The median DFS of 12 months was modest, however approximately 30% of patients remained progression-free at 4 years. Loco-regional recurrence was the main cause of death, again demonstrating that recurrent disease is difficult to control, both at first and subsequent relapse.

In a previous study, 130 patients who underwent salvage surgery were randomly assigned to receive reirradiation (60 Gy) combined with concomitant chemotherapy (5-FU and hydroxyurea) versus no adjuvant treatment [12]. A significant improvement with regard to the primary endpoint of loco-regional tumour control (hazard ratio (HR) 2.7; 95% confidence interval (CI) 1.7–4.5; *p* < 0.001) and DFS (HR 1.7; 95% CI 1.1–2.5; *p* = 0.01) was observed in those patients who were assigned to receive postoperative chemo-reirradiation compared to those who underwent surgery alone. However, this benefit in DFS did not translate into a significant improvement of overall survival. The gain in loco-regional tumour control and DFS was achieved at the cost of significantly higher rates of grade 3–4 late side effects (39% versus 10% at 2 years, respectively). Importantly, patients allocated to the wait-and-see arm could receive salvage chemo-reirradiation at the time of loco-regional recurrence after salvage surgery, which was the case in 25% of patients. This type of cross-over reduces the likelihood of improved overall survival. In the newer but smaller hyperfractionation study [21], 2/14 patients in that arm had grade 3–4 toxicity at 2 years (trismus and dysphagia), supporting the concept of hyperfractionation if one proceeds to postoperative reirradiation in selected cases. Based on the results of both studies, a wait-and-see policy may also be considered, in particular when patients suffer from continuous late toxicity from the first course of treatment.

### Primary brain tumours

Bergman et al. performed a small, prematurely closed trial of fractionated stereotactic radiosurgery with chemotherapy versus chemotherapy alone for bevacizumab (BEV)-resistant high-grade glioma, largely glioblastoma [22]. Chemotherapy drugs were chosen at the discretion of the treating physician recommended by the tumor board. Patients were stratified by KPS (≤ 80 vs > 80). The primary endpoints were local tumour control at 2 months and PFS. It was assumed that the reirradiation group would have a local control rate of 40% compared to a 10% rate in the comparator group. A total dose of 32 Gy (4 fractions of 8 Gy) was prescribed to a gross tumour volume (GTV; range 3‑186 cc), defined as the T1-weighted contrast-enhancing lesion plus the area of diffusion-weighted imaging seen on the co-registered magnetic resonance imaging scans (MRI). A dose of 24 Gy was prescribed to a clinical target volume (CTV) defined as the area of the new or change in T2-weighted fluid-attenuated inversion recovery abnormality. Treatment was planned via a simultaneous integrated boost (SIB) technique. The dose was prescribed to the highest isodose line encompassing the CTV, which ranged from 50 to 95% of the maximum dose. Planning target volume (PTV) was equal to GTV or CTV, respectively. There was no limit to the maximum target volume. It was assumed that all critical structures had received the maximum point dose during initial external beam radiation treatment. The dose constraints from the American Association of Physicists in Medicine Task Group 101 (TG101) report of stereotactic body radiation therapy for 3‑fraction treatments were used [30]. Coverage to the 32 Gy volume was not compromised based on those constraints. The median time from initial diagnosis to enrollment was 20.5 months (range 7 to 268) and the median number of prior recurrences was 3 (range 2 to 6). The reirradiation group had an improved median PFS compared to BEV-based chemotherapy alone (5.1 months, 95% CI 4.1–6.2 vs 1.8 months, 95% CI 1.2–2.8; *p* < 0.001). The reirradiation group had a better overall survival compared to the BEV/chemotherapy only group, but this difference was not statistically significant (median overall survival: 7.2 months [95% CI 6.1–8.1] vs 4.8 months [95% CI 1.7–7.6]; *p* = 0.11). Reirradiation toxicity grade 3 included one patient each with headache, nausea/vomiting, new onset weakness, intratumoural hemorrhage, and seizure (no grade 4 or 5 toxicities). There were no documented cases of radionecrosis.

Lower reirradiation EQD2 was employed in the German ERGO2 trial, a randomized trial of calorie-restricted ketogenic diet and fasting in addition to reirradiation for malignant glioma, largely glioblastoma [23]. It included 50 patients and required KPS ≥ 60 and interval at least 6 months, among other criteria. Most patients had 5 or 10 fractions of reirradiation (5 fractions of 4 Gy (mainly), 10 fractions of 3.5 Gy). The authors assumed PFS at 6 months to increase from 0% to 30%. No significant difference was observed in PFS at 6 months: 20% vs. 16%, *p* = 0.7. Similar median overall survival was achived (10.9 vs. 9.5 months). During the dietray intervention phase until day 12, 9 adverse events (experimental arm: 4, standard diet (SD): 5) were reported. Three patients suffered from epileptic seizures. The other adverse events were headache, nausea, or possible epileptic seizures with short-lasting aphasia, which could not be categorized by the description from the patient. From day 12 until the first MRI follow-up after 1 month, 11 adverse events (experimental arm: 5, SD: 6) were reported, the majority of which were epileptic seizures. The publication did not comment on radionecrosis. In summary, ERGO2 demonstrated that this dietary intervention can be safely applied to patients with recurrent glioma.

The largest (*n* = 170) and thus most important recent trial was NRG Oncology/RTOG1205, a randomized phase 2 trial to determine whether BEV plus reirradiation (experimental arm) would improve survival (primary endpoint) compared with BEV alone (control arm) [24]. Patients were stratified by age (< 50 years vs ≥ 50 years), KPS (60 vs 70–80 vs 90–100), and recent re-resection. Inclusion criteria were modified after slow accrual to allow for patient enrollment with up to three relapses, a KPS of ≥ 60, and recurrent tumors ≤ 6 cm. Multifocal recurrence was no longer excluded, provided that the composite tumor volume was ≤ 6 cm. Reirradiation dose was 35 Gy in 10 fractions, using 3D conformal technique, IMRT or protons. The protocol defined certain organ-at-risk doses (planning organ-at-risk volumes, 3 mm margin) and acceptable variations. Optic nerves and chiasm (D0.3cc) were constrained to 20 Gy (acceptable variation: 25 Gy). The respective figures were 24 and 30 Gy for brain stem (D0.3cc). GTV was defined as enhancing tumour using computed tomography and/or MRI or postoperative resection cavity if no residual enhancing tumour was noted. A PTV expansion of at least 3 mm was used. BEV was administered at a dose of 10 mg/kg every 2 weeks until disease progression. Patients randomly assigned to the BEV and reirradiation arm received an initial induction BEV dose (day 1) followed by concurrent BEV and radiation at the next dose (day 14), and then once every 14 days until disease progression. The median survival for the control arm was 9.7 months (80% CI, 9.0 to 11.2) and 10.1 months (95% CI, 9.5 to 11.3) for the experimental arm (HR, 0.98; 80% CI, 0.79 to 1.23, *p* = 0.46). Twelve patients on the BEV arm received reirradiation as salvage therapy. Furthermore, some imbalances in baseline characteristics favoured the BEV arm. The only notable survival difference between arms was noted for the KPS 90–100 subgroup, in which the BEV/reirradiation arm showed improved survival (HR, 0.67; 95% CI, 0.40 to 1.13; *p* = 0.13). The median PFS for the control versus reirradiation arms was 3.8 versus 7.1 months, respectively (HR, 0.73; 95% CI, 0.53 to 1.00; *p* = 0.05). No delayed grade 3 or worse treatment-related central nervous system adverse events were reported. The authors discussed that optimal treatment for patients with recurrent glioblastoma remains controversial in the absence of improved survival. Their study confirmed meaningful improvement in PFS, including the 6‑month PFS rate, which patients may consider clinically beneficial. Quality-of-life was not evaluated. Treatment was safe and well-tolerated with no delayed brain toxicities. Therefore, reirradiation (the study regimen or fewer stereotactic fractions) remains a reasonable option especially for patients with small volume of recurrence and good KPS, as also reflected in a recent guideline [31].

### Breast cancer

Schouten et al. reported a prematurely closed randomized phase 2 study of reirradiation and hyperthermia versus reirradiation and hyperthermia plus chemotherapy for locally recurrent breast cancer in previously irradiated areas not suitable for resection (*n* = 49, planned: 104, slow accrual) [25]. Concurrent hormonal therapy was allowed. Patients were stratified by size of recurrence (> 5 cm or ≤ 5 cm) and time interval between primary breast cancer and first recurrence (> 3 years or ≤ 3 years). The authors tried to detect an increase in the local control rate after 1 year from 54% in the standard treatment arm to 69% in the experimental arm (corresponding to a HR of 0.6). Originally, 32 Gy was given in 8 fractions of 4 Gy in 4 weeks, at 2 fractions per week (3 days in between the fractions). After January 2015, the radiotherapy schedule was changed to 46 Gy in 23 fractions of 2 Gy, at 5 fractions per week. Local microwave hyperthermia was delivered once a week, starting within 1 h after radiotherapy. Patients receiving the 32-Gy schedule were given four sessions of hyperthermia and patients receiving the 46-Gy schedule were given five sessions of hyperthermia, a modality with long track record [32, 33]. Patients were treated with weekly cisplatin 40 mg/m^2^ given intravenously for 4 courses, concurrent with hyperthermia. Approximately half of the patients had already been unsuccessfully treated for the current relapse with surgery, chemotherapy, hormonal therapy or trastuzumab. Local (infield) progression-free rate at 1 year was high in both arms, 81.5% in the standard arm and 88% in the combined arm. About 60% per arm achieved a complete response. With or without cisplatin, most patients had subsequent local control until last follow-up or death. No significant difference regarding any endpoint was observed. One patient in the standard arm died due to a necrotizing thoracic wall defect in the radiated area 3 months after treatment. We could not identify randomized trials on repeat breast-conserving surgery with reirradiation, a concept gaining increasing acceptance in well-selected patients [34].

## Summary and conclusions

The objective of this follow-up study was to review all recently published randomized trials in order to identify methodological strengths and weaknesses, comment on the results and open questions, and highlight the role of the upcoming Recare trial in collecting cumulative dose distributions through a new cohort within the E2-RADIatE platform managed by the EORTC [8]. By adhering to the new consensus for trial reporting, the authors of future reirradiation publications can improve clinical practice and our understanding of dose-response relationships. Important findings from the current review include that many trials failed to recruite as anticipated, resulting in premature closure or major protocol changes introducing heterogeneity, and eventually small group size. Nevertheless, several adequately designed, conducted and reported trials confirm again that high-level evidence can be generated in the field of reirradiation. Multi-institutional collaboration is encouraged to complete sufficiently large trials. The trials with relatively long median follow-up and high cumulative total doses confirmed that serious toxicity remains a concern, and that curative reirradiation can either prevent or cause a fatal outcome. Hyperfractionation improves the therapeutic ratio in the scenarios reviewed here. For many other clinical scenarios where reirradiation is offered by many institutions, randomized trials are still lacking, e.g., prostate cancer [35]. Published guidelines and consensus recommendations may guide decision-making [29, 31, 34–37]. Advanced technologies are helpful in creating highly conformal dose distributions, making us wonder about the potential of hyperfractionated proton or carbon-ion beam reirradiation. Few of the present studies published organ-at-risk dose constraints. Attempts to correlate toxicity with administered dose were lacking. This knowledge gap will hopefully be closed through the international Recare study, aiming at image fusion and co-registration to judge the cumulative dose distributions.
